# Effect of Brief Interpersonal Therapy on Depression During Pregnancy: A Randomized Clinical Trial

**DOI:** 10.1001/jamapsychiatry.2023.0702

**Published:** 2023-04-19

**Authors:** Benjamin L. Hankin, Catherine H. Demers, Ella-Marie P. Hennessey, Sarah E. D. Perzow, Mary C. Curran, Robert J. Gallop, M. Camille Hoffman, Elysia Poggi Davis

**Affiliations:** 1 Department of Psychology, University of Illinois at Urbana-Champaign, Champaign; 2 Department of Psychology, University of Denver, Denver, Colorado; 3 Department of Psychiatry, University of Colorado Anschutz Medical Campus, Aurora; 4 Division of Maternal and Fetal Medicine, Department of Obstetrics and Gynecology, University of Colorado Denver School of Medicine, Aurora; 5 School of Social Work, University of Washington, Seattle; 6 Department of Mathematics, West Chester University, West Chester, Pennsylvania; 7 Department of Pediatrics, University of California, Irvine, Irvine

## Abstract

**Importance:**

Prenatal depression is prevalent with negative consequences for both the mother and developing fetus. Brief, effective, and safe interventions to reduce depression during pregnancy are needed.

**Objective:**

To evaluate depression improvement (symptoms and diagnosis) among pregnant individuals from diverse backgrounds randomized to brief interpersonal psychotherapy (IPT) vs enhanced usual care (EUC).

**Design, Setting, and Participants:**

A prospective, evaluator-blinded, randomized clinical trial, the Care Project, was conducted among adult pregnant individuals who reported elevated symptoms during routine obstetric care depression screening in general practice in obstetrics and gynecology (OB/GYN) clinics. Participants were recruited between July 2017 and August 2021. Repeated measures follow-up occurred across pregnancy from baseline (mean [SD], 16.7 [4.2] gestational weeks) through term. Pregnant participants were randomized to IPT or EUC and included in intent-to-treat analyses.

**Interventions:**

Treatment comprised an engagement session and 8 active sessions of brief IPT (MOMCare) during pregnancy. EUC included engagement and maternity support services.

**Main Outcomes and Measures:**

Two depression symptom scales, the 20-item Symptom Checklist and the Edinburgh Postnatal Depression Scale, were assessed at baseline and repeatedly across pregnancy. Structured Clinical Interview for *DSM-5* ascertained major depressive disorder (MDD) at baseline and the end of gestation.

**Results:**

Of 234 participants, 115 were allocated to IPT (mean [SD] age, 29.7 [5.9] years; 57 [49.6%] enrolled in Medicaid; 42 [36.5%] had current MDD; 106 [92.2%] received intervention) and 119 to EUC (mean [SD] age, 30.1 [5.9] years; 62 [52.1%] enrolled in Medicaid; 44 [37%] had MDD). The 20-item Symptom Checklist scores improved from baseline over gestation for IPT but not EUC (*d* = 0.57; 95% CI, 0.22-0.91; mean [SD] change for IPT vs EUC: 26.7 [1.14] to 13.6 [1.40] vs 27.1 [1.12] to 23.5 [1.34]). IPT participants more rapidly improved on Edinburgh Postnatal Depression Scale compared with EUC (*d* = 0.40; 95% CI, 0.06-0.74; mean [SD] change for IPT vs EUC: 11.4 [0.38] to 5.4 [0.57] vs 11.5 [0.37] to 7.6 [0.55]). MDD rate by end of gestation had decreased significantly for IPT participants (7 [6.1%]) vs EUC (31 [26.1%]) (odds ratio, 4.99; 95% CI, 2.08-11.97).

**Conclusions and Relevance:**

In this study, brief IPT significantly reduced prenatal depression symptoms and MDD compared with EUC among pregnant individuals from diverse racial, ethnic, and socioeconomic backgrounds recruited from primary OB/GYN clinics. As a safe, effective intervention to relieve depression during pregnancy, brief IPT may positively affect mothers’ mental health and the developing fetus.

**Trial Registration:**

ClinicalTrials.gov Identifier: NCT03011801

## Key Points

**Question:**

Can depression be reduced during pregnancy and before birth using a brief, safe intervention?

**Findings:**

In this randomized clinical trial of 234 adult pregnant individuals, elevated symptoms were reported during routine obstetric care depression screening. Compared with enhanced usual care, brief interpersonal psychotherapy significantly improved depression symptoms and major depressive disorder diagnosis rate during pregnancy.

**Meaning:**

Brief interpersonal psychotherapy may be warranted to relieve depression in pregnant individuals with potential intergenerational benefits.

## Teaser

This randomized clinical trial evaluates depression improvement (symptoms and diagnosis) among pregnant individuals from diverse backgrounds randomized to brief interpersonal psychotherapy vs enhanced usual care.

## Introduction

Depression is common and contributes to disability and disease burden.^1^ Approximately 17% of pregnant individuals meet criteria for major depressive disorder (MDD) diagnosis,^2^ and up to 37% report elevated symptoms during pregnancy.^3^ Prenatal maternal depression confers intergenerational risks, including preterm birth as well as developmental delays, and enhanced vulnerability to psychopathology in offspring.^4,5,6,7,8,9,10^ The Perinatal Depression Task Force of the American College of Obstetricians and Gynecologists^11^ highlighted the need for early screening of depression and intervention during pregnancy. Still, most published work and current health care policy has emphasized preventing postpartum depression with scant attention focused on reducing prenatal depression.^12^ With pregnancy as a sensitive period of enhanced vulnerability for both pregnant individuals and the developing fetus, we identified a clear need for a randomized clinical trial (RCT) implemented exclusively during pregnancy with the goal of evaluating a brief, safe intervention to decrease prenatal depression.

The US Preventive Services Task Force (USPSTF) showed that current interventions exhibit significant but small effects to prevent perinatal depression (defined broadly including pregnancy through 12 months post partum).^12^ This recent USPSTF systematic review summarized perinatal counseling interventions and noted several limitations of the literature. They advocated for larger-scale effectiveness trials that use good-quality design, assess depression repeatedly throughout pregnancy with dimensional symptom measures, and focus on individuals at elevated risk for depression recruited from general primary obstetrics and gynecology (OB/GYN) care.

The present study follows the USPSTF’s recommendations for investigating psychosocial interventions^12^ with few to no adverse effects on fetal development. The efficacy of interpersonal psychotherapy (IPT) has been evaluated previously with pregnant^13,14,15,16^ and postpartum^17,18,19^ participants, and these existing trials support the need for well-powered studies assessing the effect of IPT on depression within the prenatal period. There is a critical need to reduce depression within the prenatal period because both depression diagnosis and dimensional symptom elevations are risks for important health outcomes, including obstetric complications, preterm birth, decreased likelihood of breastfeeding, and child developmental delays.^20,21,22,23^ Thus, reducing depression prenatally can benefit maternal health (eg, risk for postpartum depression) and improve offspring outcomes given links between prenatal depression and childhood developmental outcomes (eg, brain development, stress regulation, and psychopathology^5,6,24,25,26,27^). We report data from this RCT (the Care Project) in which we compared an evidence-based brief IPT (MOMCare^28,29,30^) with enhanced usual care (EUC).

## Methods

### Participants

We randomized 234 pregnant individuals recruited primarily from obstetrics clinics at 2 major medical centers in the Denver, Colorado, metropolitan area. Recruitment began July 2017 and ended August 2021 with a pause from mid-April through mid-June 2020 due to the COVID-19 pandemic. Initial screening was performed based on medical record review. Eligibility criteria included 18 to 45 years of age, English speaking, 25 weeks’ gestational age (GA) or less, singleton pregnancy, and endorsing elevated depression symptoms when screened as part of standard of care (Edinburgh Postnatal Depression Scale [EPDS] score ≥10). Exclusion criteria included current illicit drug or methadone use and major health conditions requiring invasive treatments (eg, dialysis, blood transfusions, chemotherapy). Additional assessment for eligibility was performed at baseline to exclude (1) current or past psychosis or mania and (2) currently receiving cognitive behavioral therapy or IPT. The trial protocol is available in Supplement 1. Institutional review boards for the Protection of Human Subjects at the University of Denver and the University of Colorado Anschutz Medical Campus approved the study. Recruitment, outcomes, and adverse events were monitored by a data and safety monitoring board. Participants provided written and informed consent.

**Figure 1.**
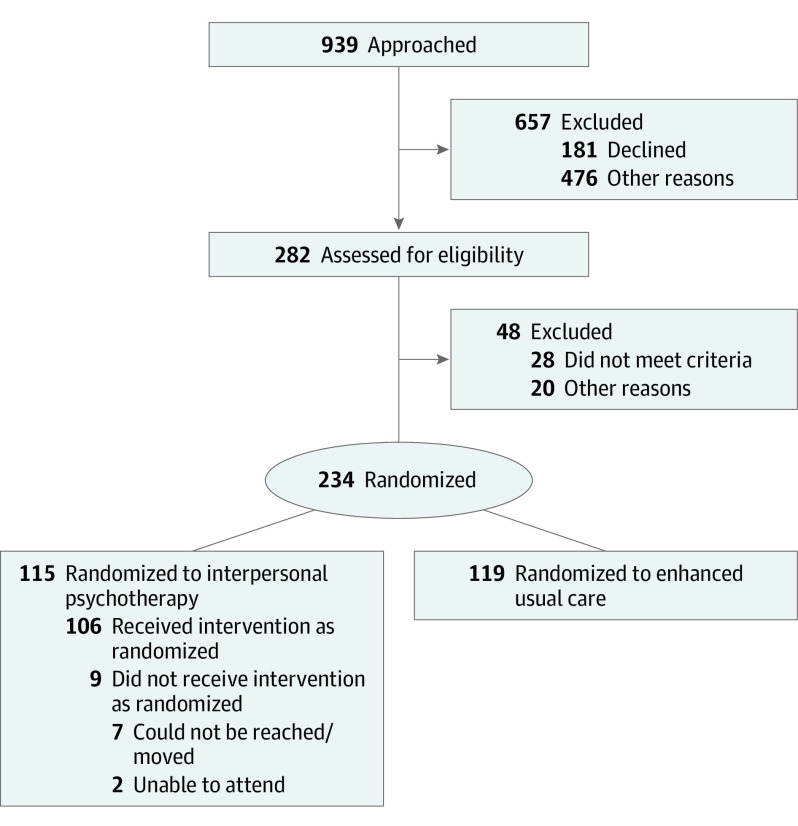
CONSORT Flow Diagram Participants allocated to interpersonal psychotherapy and designated as “did not receive intervention” did not attend any prenatal intervention sessions.

This study followed the Consolidated Standards of Reporting Trials (CONSORT) reporting guideline. Figure 1 provides the CONSORT diagram and illustrates allocation to intervention condition and participant flow (eMethods 1 in Supplement 2). Individuals were allocated to EUC or IPT. Participants (including 9 receiving no intervention) were considered part of the study once randomized (intent-to-treat design).

### Procedures

Participants completed a baseline evaluation, including interview and questionnaire assessments. Eligible individuals were then randomly assigned to IPT or EUC using a computer-generated random numbers sequence. Participants were randomized in blocks of 2 and stratified on current MDD (assessed via the Structured Clinical Interview for *DSM-5* [SCID-5]) based on *DSM-5* criteria, GA (above or below 15 weeks), and Medicaid status for each study arm. Participants were compensated for completing study measures (via REDcap or with blinded evaluator) 3 to 5 times prenatally: (1) baseline: mean (SD) of 16.7 (4.2) gestational weeks (prior to intervention), (2) 22.3 (4.1) weeks, (3) 25.7 (4.1) weeks, (4) 29.7 (4.2) weeks (near end of active IPT), and (5) 35.8 (1.5) weeks (end of gestation). An overview of the study timeline is shown in the eFigure in Supplement 2.

#### Intervention Conditions

MomCare is a culturally relevant, collaborative care intervention that provides brief IPT.^30^ Before starting IPT sessions, study therapists implemented a manualized ethnographic session^30^ to engage pregnant individuals, sustain treatment participation, and help resolve practical, cultural, and psychological barriers to care. Brief IPT consists of eight 50-minute individual sessions, approximately a week apart, with the active phase of treatment during pregnancy; thereafter, maintenance care is allowed with less frequent sessions.^31^ Intervention focuses on psychoeducation and interpersonal skill building to decrease interpersonal conflict and increase interpersonal support and competence. Individuals are educated about the link between feelings and interpersonal interactions and learn strategies to resolve interpersonal conflicts contributing to depression symptoms (eAppendix in Supplement 2). MOMCare includes key elements of collaborative care. Participants were provided community support resources and could be prescribed antidepressant medications.

All IPT therapists and collaborative care team members completed a 3-day training and were certified by Nancy Grote, PhD, a leading brief IPT expert for antenatal depression. IPT therapists were doctoral-level clinicians who followed detailed treatment manuals and received supervised training. Adherence checklists ensured that MOMCare was delivered with fidelity. Fidelity ratings were very high (mean, 1.90; scale ranged from 0 [needs work] to 2 [done well]) across sessions independently rated by MOMCare developers Nancy Grote, PhD, and Mary C. Curran, MSW. All participants had at least 1 session reviewed; 25% to 40% of sessions across active IPT were reviewed (eMethods 2 in Supplement 2). Most participants attended nearly all 8 prenatal IPT sessions (mean [SD], 7.27 [3.5]).

EUC augmented the usual standard of care pregnant individuals received within OB/GYN clinics through their obstetric clinicians and/or social workers. EUC consisted of maternity support services, which provides mental health counseling integrated within the obstetric setting and 1-on-1 consultation session with doctoral-level clinicians. Through maternity social services, pregnant individuals were offered mental health support, based on patient preferences, including prepartum depression care (eclectic counseling [not IPT or cognitive behavioral therapy] or psychiatric consultation including medication) and other community services. During this 1-on-1 sixty-minute consultation session, the clinician reviewed written psychoeducational materials on perinatal depression so participants could better recognize mood symptoms during pregnancy and discussed with the participant ways to talk with partners or other supports about depression to elicit help. Clinicians conducted ongoing monitoring with participants and provided referrals to patients to overcome barriers and when care was needed. Extensive community resources were provided, including information on additional alternative community mental health and counseling services, essential items and housing programs, and local resource centers for pregnancy and postpartum support. Overall, 58 participants (48.8%) reported using additional counseling. EUC occurred during the same time frame as MOMCare.

#### Measures and Outcomes

The 20-item Symptom Checklist (SCL-20) measures depression symptoms from the full Symptom Checklist-90-R.^32^ Items are rated on a scale of 0 to 4 and summed to generate a total score, ranging from 0 to 80. Higher ratings indicate more depression. Scores below 20 suggest MDD remission.^33^ Prior work indicates reliability and validity, including with pregnant participants.^34^ Internal consistency was excellent (α = .91 at all 3 time points: baseline, 28 gestational weeks, and 35 gestational weeks).

EPDS is used to screen maternal depression across the peripartum period. Research shows reliability and validity in pregnancy.^35,36^ Participants provided ratings on a scale of 0 to 3. Higher scores signify greater depression. Total scores range from 0 to 30. Scores of 10 or higher suggest probable depression.^36^ Internal consistencies ranged from α = 0.82 to 0.89 across 5 time points.

Prior to randomization, trained independent evaluators administered the SCID-5 at baseline to determine participants’ diagnostic status using *DSM-5* criteria, including MDD and the exclusion criteria of mania or psychosis (current or lifetime). Additionally, independent evaluators interviewed participants post partum to ascertain MDD status at the end of pregnancy including the 2 weeks prior to delivery. Interviewers were highly reliable: κ ≥ 0.95 for MDD based on reliability review of 50% of SCIDs randomly selected.

#### Sociodemographic and Obstetric Characteristics

Research staff blinded to condition collected sociodemographic and obstetric characteristics. Birth date, socioeconomic status, cohabitation with partner, marital status, educational attainment, race (categories: American Indian/Alaska Native, Asian, Black or African American, European, Middle Eastern, Native Hawaiian or Other Pacific Islander, North African, White, or more than 1 race), and ethnicity (Chicana, Hispanic, Latine, or Mexican) were collected via interview. Income to needs ratio was calculated by dividing the total reported household income for the past year by the poverty threshold for that year corresponding to the number of persons living in the household, specified by the US Census Bureau. Two research staff performed medical record reviews, which were assigned by an independent staff member with 50% overlap in records reviewed to ensure consistency across the 2 reviewers. GA was calculated from medical record review using early ultrasonography measures and/or date of last menstrual period applying the American College of Obstetricians and Gynecologists guidelines.^37^

### Data Analytic Strategy

All models were fit using SAS statistical software version 9.4 (SAS Institute). All analyses were conducted with the full intention-to-treat sample. We compared differences by condition on baseline demographic and clinical characteristics using *t *tests and χ^2^ tests for continuous and categorical variables, respectively.

To test hypotheses that participants assigned to IPT would experience reduced depression symptoms across pregnancy, we implemented hierarchical linear modeling^38^ to accommodate within-participant correlation over time for SCL-20 and EPDS. With time defined as weeks after randomization, linear change best described change trajectories for these outcomes over time (eResults 1 in Supplement 2). We used Kenward-Roger approximation^39^ to estimate degrees of freedom. Effect sizes by intervention group for degree of change in depression symptoms (SCL-20 and EPDS) across pregnancy were estimated as Cohen *d*.^40^ For the dichotomous outcome of MDD diagnosis by end of gestation, logistic regression was used with odds ratio (OR) as effect size. GA at randomization and baseline MDD were tested as moderators (eResults 2 in Supplement 2).

Statistical power was calculated for the between-groups design (IPT vs EUC) using estimates based on prior IPT perinatal depression intervention studies.^13,14,15,16,17,18,19,28,30,31^ A priori power was estimated to be 99% with the initially proposed sample size of 256 to detect a moderate effect (*d* = 0.50). The observed sample size (n = 234) was slightly lower than planned (n = 256) due to recruitment pause during the COVID-19 pandemic. Power was above 95% to detect this same moderate effect with the observed sample. Two-sided *P* values were statistically significant at α = .05.

## Results

### Preliminary Analyses

Of 234 individuals, 119 were allocated to EUC and 115 assigned to IPT (106 of whom received intervention, and 9 participated in no intervention sessions). Participants reported their race and ethnicity as 10 (4.3%) Asian, 21 (9%) Black, 43 (18.4%) Latine, 1 (0.4%) Native Hawaiian or Pacific Islander, 101 (43.2%) non-Hispanic/Latine White, and 58 (24.8%) multiracial or multiethnic (Table 1). The mean (SD) age was 29.8 (5.9) years at recruitment (range, 18 to 42 years). The median (IQR) annual household income was $50 000 ($25 000%-90 000) (equivalent median for Denver is $72 661 per 2020 census^41^), and 95 participants (40.4%) were living at or near federal classification of poverty (less than 200% income to needs ratio). Participants reported on psychotropic medication use during pregnancy (IPT: 21 [22.3%] and EUC: 29 [28.2%]; χ^2^ = 0.88; *P* = .35). Table 1 provides sample characteristics delineated by IPT vs EUC group.

**Table 1.** Descriptive Information for the Study Sample

Characteristic	No. (%)		Effect size (95% CI)^a^
	IPT group	EUC group	
No.	115	119	NA
Maternal age at recruitment, mean (SD)	29.7 (5.9)	30.0 (5.9)	0.06 (−0.20 to .31)
Gestational age at recruitment, mean (SD), wk	16.9 (4.5)	16.5 (3.9)	0.05 (−0.21 to .30)
Parity (nulliparous)	61 (53.0)	47 (39.5)	1.73 (1.03 to 2.91)
Race and ethnicity			
Asian	6 (5.2)	4 (3.4)	1.58 (0.44 to 5.76)
Black	11 (9.6)	10 (8.4)	1.15 (0.47 to 2.83)
Hispanic or Latine	19 (16.5)	24 (20.2)	0.78 (0.40 to 1.52)
Native Hawaiian/Pacific Islander	1 (0.9)	0	1.00 (0.99 to 1.03)
Non-Latine White	49 (42.6)	52 (43.7)	0.96 (0.57 to 1.61)
Multiracial or multiethnic	29 (25.2)	29 (24.4)	1.05 (0.58 to 1.89)
Annual household income, median (IQR), $	47 000 (25 000-88 500)	50 000 (25 450-95 000)	0.06 (−0.20 to 0.31)
Household income to needs ratio (living below 200% of federal poverty line)	44 (38.3)	49 (41.2)	0.89 (0.52 to 1.50)
Medicaid status (enrolled)	57 (49.6)	62 (52.1)	0.90 (0.54 to 1.51)
Cohabitating with partner	85 (73.9)	89 (74.8)	0.96 (0.53 to 1.72)
Education (highest degree earned)			
<High school	6 (5.2)	7 (5.9)	0.88 (0.29 to 2.70)
High school	24 (20.9)	21 (17.6)	1.23 (0.64 to 2.36)
Some college	33 (28.7)	37 (31.1)	0.89 (0.51 to 1.56)
College degree	33 (28.7)	36 (30.2)	0.93 (0.53 to 1.63)
Graduate degree	19 (16.5)	18 (15.2)	1.11 (0.55 to 2.24)
Depression diagnosis			
MDD at recruitment	42 (36.5)	44 (37.0)	0.98 (0.58 to 1.67)
MDD end of gestation	7 (6.1)	31 (26.1)	4.99 (2.08 to 11.97)

Abbreviations: EUC, enhanced usual care; IPT, interpersonal psychotherapy; MDD, major depressive disorder (diagnosis per Structured Clinical Interview using *DSM-5* criteria); NA, not applicable.

^a^ Effect sizes for continuous measures are Cohen *d,* and binary measures are odds ratio, specifically the odds for IPT compared with EUC.

Retention rates through prenatal assessments were 88% (101 of 115) in IPT and 87% (104 of 119) in EUC (χ^2^_1_ = 0.01; *P* = .92). Model-based residuals indicated no deviation in multivariate normality (Shapiro-Wilk test^42^ ranging from 0.97-0.99 per assessment point for EPDS and 0.97-0.98 for SCL-20). Pattern-mixture model^43^ results indicated that missing data patterns were not driving intervention effects on EPDS (*F*_1,225_ = 2.09; *P* = .15) or SCL-20 (*F*_1,225_ = 1.99; *P* = .16). All observed data were used to create a complete data set via Markov chain Monte Carlo imputation methods^44^; this complete data set was used for analyses. Parity was the only demographic/obstetric factor that significantly differed between groups, so analyses included parity as a covariate. There were no significant therapist or site effects (eResults 3 in Supplement 2).

### Intervention Effects

#### SCL-20

**Figure 2.**
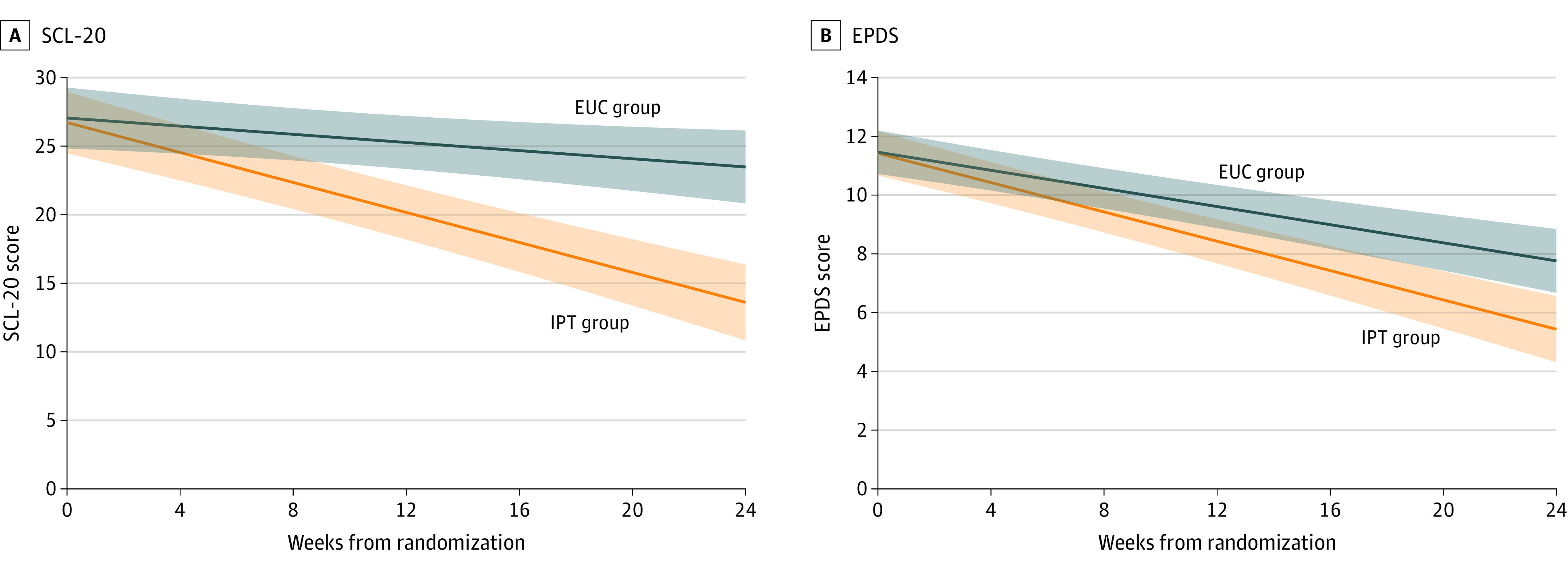
Effect of Intervention on Depression Symptom Trajectories Over Pregnancy The color shaded regions represent 95% CIs. Hierarchical linear modeling (HLM) results with full sample for intent-to-treat analyses included 115 individuals in the interpersonal psychotherapy (IPT) group and 119 in the enhanced usual care (EUC) group. A, Depressive symptoms were measured by the 20-item Symptom Checklist (SCL-20) and showed significant intervention × time interaction (*t*_229_ = 4.31; *P* < .001; *d* = .57) with differential improvement for IPT relative to EUC on SCL-20 depression symptoms over time. SCL-20 scores can range up to 80. B, Depressive symptoms were measured by the Edinburgh Postnatal Depression Scale (EPDS). HLM showed significant intervention × time interaction (*t*_229_ = 2.73; *P* = .007; d = .40) with differential improvement for IPT relative to EUC on EPDS depression symptoms over time.

There was a differential rate of change in depression symptoms assessed via SCL-20 between IPT and EUC from randomization through the end of gestation (*t*_229_ = 4.31; *P* < .001; Table 2). Participants assigned to IPT showed a mean (SE) point reduction of 0.551 (0.067) in SCL-20 scores per week compared with EUC (mean [SD] reduction, 0.149 [0.065]); medium effect size (*d* = 0.57; 95% CI, 0.22-0.91). Figure 2A shows the IPT group on average exhibited a mean (SE) of 0.402 (0.093) points greater reduction per week compared with EUC; IPT significantly separated from EUC by 6 to 7 weeks postrandomization.

**Table 2.** Model-Based Estimated Depression Symptoms Over Time for IPT and EUC

Depressive symptoms	Mean (SE)^a^		Between-group test of statistical significance	Cohen *d* effect size (95% CI)
	IPT group	EUC group		
No.	115	119		
SCL-20^b^				
Week 0	26.72 (1.14)	27.05 (1.12)	*t* *= *0.11, *P* = .91	0.01 (−0.21 to 0.27)
Week 4	24.53 (1.03)	26.45 (1.02)	*t* *= *1.22, *P* = .22	0.16 (−0.03 to 0.33)
Week 8	22.34 (0.98)	25.86 (0.97)	*t* = 2.43, *P* = .02	0.32 (0.06 to 0.58)
Week 12	20.16 (1.00)	25.26 (.098)	*t* *= *3.52, *P* = .001	0.46 (0.20 to 0.72)
Week 16	17.97 (1.08)	24.67 (1.05)	*t* *= *4.29, *P* < .001	0.57 (0.31 to 0.83)
Week 20	15.78 (1.22)	24.07 (1.18)	*t* = 4.74, *P* < .001	0.63 (0.37 to 0.89)
Week 24	13.6 (1.40)	23.48 (1.34)	*t* *= *4.96, *P* < .001	0.66 (0.40 to 0.92)
EPDS^c^				
Week 0	11.42 (0.38)	11.45 (0.37)	*t* = 0.07, *P* = .95	0.01 (−0.25 to 0.27)
Week 4	10.42 (0.35)	10.84 (0.35)	*t* *= *0.83, *P* = .41	0.11 (−0.15 to 0.37)
Week 8	9.42 (0.35)	10.22 (0.35)	*t* = 1.59, *P* = .11	0.21 (−0.05 to 0.47)
Week 12	8.42 (0.38)	9.60 (0.37)	*t* = 2.19, *P* = .03	0.29 (0.03 to 0.55)
Week 16	7.42 (0.43)	8.99 (0.42)	*t* = 2.58, *P* = .01	0.34 (0.08 to 0.60)
Week 20	6.42 (0.49)	8.37 (0.48)	*t* = 2.80, *P* = .006	0.37 (0.11 to 0.63)
Week 24	5.43 (0.57)	7.75 (0.55)	*t* = 2.92, *P* = .004	0.39 (0.13 to 0.65)

Abbreviations: EPDS, Edinburgh Postnatal Depression Scale; EUC, enhanced usual care; IPT, interpersonal psychotherapy; SCL-20, 20-item Symptom Checklist.

^a^ Mean estimates and standard error derived from the fitted hierarchical linear modeling adjusted for covariates and the variance-covariance structure. Test of significance are based on linear contrasts at respective time within each hierarchical linear modeling model. Week 0 = time of randomization.

^b^ SCL-20 scores below 20 are consistent with no major depressive disorder diagnosis.^33^

^c^ EPDS scores at or below 9 are consistent with no major depressive disorder diagnosis.^36^

#### EPDS

A differential rate of change over time between IPT and EUC groups was observed for EPDS (*t*_229_ = 2.73; *P* = .007; Table 2). Specifically, participants assigned to IPT had a steeper reduction in symptoms with a mean (SE) decrease of 0.250 (0.025) per week, compared with EUC (mean [SE] reduction of 0.154 [0.026] per week). Figure 2B shows this medium effect (*d* = 0.40; 95% CI, 0.06-0.74). On average, the IPT group showed a mean (SE) of 0.095 (0.035) points reduction per week compared with EUC, resulting in IPT significantly differing from EUC by 11 weeks postrandomization.

#### SCID-5 MDD

**Figure 3.**
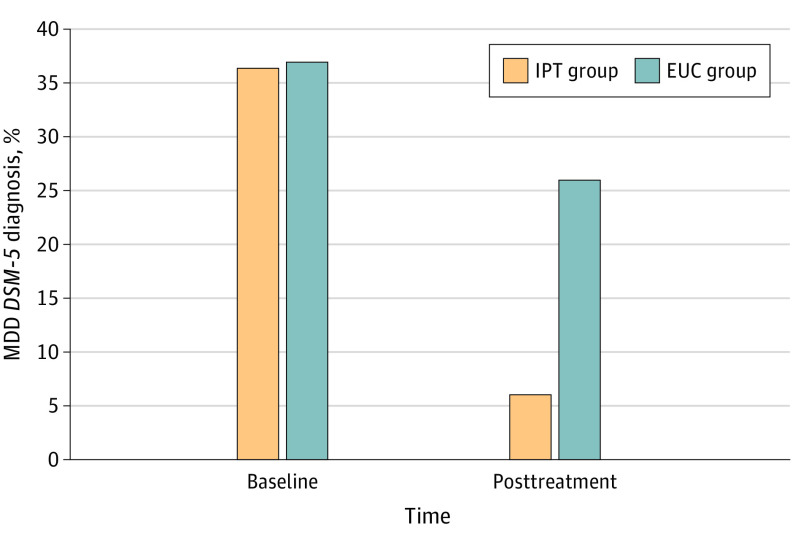
Effect of Intervention on Major Depressive Disorder (MDD) *DSM-5* Diagnosis Rate With full sample for intent-to-treat analyses (interpersonal psychotherapy [IPT]: n = 115; enhanced usual care [EUC]: n = 119), between-groups analysis showed significant difference (χ^2^_1_ = 17.13; *P* < .001) for *DSM-5* MDD rate posttreatment (end of gestation). Participants assigned to IPT showed greater MDD remittance relative to EUC (odds ratio, 4.99; 95% CI, 2.08-11.97).

Figure 3 shows MDD rates declined from baseline to the end of gestation when rates significantly differed between IPT (7 of 115 [6.1%]) and EUC (31 of 119 [26.1%]) (OR, 4.99 [95% CI, 2.08-11.97]; χ^2^_1_ = 17.13; *b* = 1.61 [SE = 0.45]; *P* < .001; Wald = 12.97) after controlling for parity as covariate (OR, 1.38 [95% CI, 0.98-1.91]; *b* = 0.32 [SE = 0.16]; *P* = .05; Wald = 3.76).

### Moderator Analyses

We evaluated baseline MDD and GA at randomization as potential a priori moderators of treatment effects on primary study outcomes. Neither baseline MDD (SCL-20: *F*_1,229_ = 1.05, *P* = .31; EPDS: *F*_1,229_ = 0.02, *P* = .90; end of gestation MDD: Wald = 0.14, *P* = .71) nor GA (SCL-20: *F*_1,229_ = 2.38, *P* = .13; EPDS: *F*_1,229_ = 0.54, *P* = .46; end of gestation MDD: Wald = 0.02, *P* = .87) moderated treatment effects. Post hoc exploratory analyses showed that the COVID-19 pandemic did not moderate primary outcome effects (SCL-20: *t*_229_ = −0.77, *P* = .41; EPDS: *t*_229_ = −0.31, *P* = .75; eResults 4 in Supplement 2). Post hoc sensitivity analyses exploring whether psychiatric medication use affected depression symptom outcomes for participants receiving IPT showed no moderation (SCL-20: *t*_229_ = 0.21, *P* = .76; EPDS: *t*_229_ = −0.60, *P* = .55; eResults 5 in Supplement 2).

## Discussion

There is critical need to reduce depression during pregnancy given consequences for both mother and developing fetus. We initiated the Care Project to tackle this unaddressed challenge of decreasing depression for pregnant individuals with elevated depression (MDD and symptoms). Study findings demonstrated that a safe, prenatal intervention (MOMCare, brief IPT) substantially reduced depression symptoms and led to a considerable reduction in MDD diagnosis relative to pregnant individuals receiving EUC from general OB/GYN clinics.

Results of this RCT with pregnant individuals, recruited for exhibiting elevated depression symptoms at standard OB/GYN screening, showed that brief IPT significantly decreased depression, including symptoms (*d* = 0.57 for SCL-20, *d* = 0.40 for EPDS) and MDD (OR, 4.99), during the prenatal period compared with EUC. The benefit of IPT resulted in significant improvement observed relatively quickly: for example, significant between-groups difference on SCL-20 depression symptoms was noted by 6 to 7 weeks postrandomization, corresponding to symptom relief by 24 weeks’ GA on average. Brief IPT considerably reduced participants receiving MDD diagnosis by end of gestation (IPT vs EUC: 6.1% vs 26.1%) after 37% rate in both groups earlier in pregnancy. Neither GA at randomization nor baseline MDD moderated intervention effects. Notably, this prenatal intervention effectively reduced depression (diagnosis and symptoms) in this racially, ethnically, and socioeconomically diverse sample benefiting both those with elevated symptoms without current MDD and those with MDD. In sum, IPT significantly relieves depression during pregnancy, a sensitive window with opportunity to benefit both maternal well-being and fetal development, and these beneficial results of brief therapy appear robust and generalizable.^13,14,15,16,17,18,19,28,30,31^

### Strengths and Limitations

Strengths of the present work enhance confidence in the findings. The USPSTF^12^ highlighted numerous limitations of past studies evaluating counseling interventions for perinatal depression and recommended future steps to address shortcomings. Consistent with their suggestions, we used a larger-scale effectiveness trial following good-quality design per USPSTF standards (their review^12^ noted only 2 studies that met criteria for good quality). In the Care Project, retention rates were high throughout pregnancy, and sample size was determined a priori via power analysis. Pregnant individuals, who were racially and ethnically diverse, were recruited from general primary OB/GYN clinics focusing on those at risk based on standard mental health screening (elevated EPDS score) administered as part of routine obstetric care. We ascertained depression via independent evaluators using reliable diagnostic interview and 2 dimensional symptom measures assessed repeatedly across pregnancy. Significant group differences, favoring IPT relative to EUC, were observed across all depression measures. Although we were unable to include both symptom measures at all time points given practical concerns for survey burden on participants, 3 assessments are sufficient to model linear symptom trajectories across pregnancy. Care Project results exhibited larger effect sizes indicating substantial depression improvement relative to the pooled average of past work (USPSTF^12^ review: *d* = 0.20), in which 5 of 17 studies showed statistically significant group differences between control and psychotherapeutic counseling intervention across the broad perinatal period.

Regarding limitations, inclusion of only English-speaking participants limits generalizability. Our intentional focus on the prenatal period precludes evaluating persistence of depression improvement after pregnancy. Further, depression symptom measures were self-reported. Objective depression changes in MDD remittance were observed using SCID-5 interview, partially addressing potential concerns with self-report.

## Conclusions

Prenatal maternal depression is one of the strongest contributors to intergenerational transmission of depression, other forms of psychopathology, and neurodevelopmental delays.^4,5,6,7,8,9,10^ However, the literature demonstrating such associations, including several impressive prospective longitudinal naturalistic studies examining potential risk mechanisms, are almost exclusively correlational. As such, these studies are “inherently limited for drawing causal conclusions” without the necessary “experimental control gained through intervention.”^45^ Given our robust findings showing substantial improvements in maternal depression during pregnancy, future research from the Care Project will follow the neonates from birth through childhood to test rigorously whether reducing depression during pregnancy affects the development of infants’ risk mechanisms for later emerging depression, anxiety, and other health problems. Grounded in the Fetal Origins of Adult Disease Hypothesis and established rapid changes in fetal brain development occurring during the prenatal period,^9^ future research can tackle whether fetal exposure to maternal depression and related prenatal stress signals affects later infant and child neurodevelopment using an RCT design.^6^

From an implementation perspective, it is encouraging that IPT reduced depression relatively quickly after starting treatment and that recipients benefited from treatment regardless of baseline MDD status or GA at randomization. Such findings inform inclusivity of care for those who do not receive early obstetric care or depression screening and show they can still benefit from brief IPT.

## Notes

Supplement 1. Trial protocol

Supplement 2. eMethods 1. Additional details on CONSORT diagram for exclusions eMethods 2. Adherence and fidelity ratings eFigure. Depiction of general Care Project RCT timeline, with the time points and study elements occurring when they tended to happen on average for modal participants eAppendix. MOMCare Vignette eResults 1. Change over time details eResults 2. Moderation eResults 3. Therapist or site effects eResults 4. COVID differences eResults 5. Sensitivity analyses for psychiatric medication use

Supplement 3. Data sharing statement
