# Supplementary material for: Effect of Brief Interpersonal Therapy on Depression During Pregnancy: A Randomized Clinical Trial
Source: JAMA Psychiatry. 2023 Apr 19;80(6):539–47. doi: 10.1001/jamapsychiatry.2023.0702 (PMC10116385; doi:10.1001/jamapsychiatry.2023.0702)
Supplement: Supplement 1. — Trial protocol [file jamapsychiatry-e230702-s001.pdf]

COMIRB Protocol

COLORADO MULTIPLE INSTITUTIONAL REVIEW BOARD
CAMPUS BOX F-490 TELEPHONE: 303-724-1055 Fax: 303-724-0990

Protocol #: 16-2040
Project Title: The Care Project: Helping Pregnant Moms and Babies
Principle Investigator: Camille Hoffman, MD
Version Date: 5-NOV-2021

Study Overview. This is a prospective longitudinal study in which we will test the hypothesis that manipulating maternal depressive symptoms during pregnancy will benefit maternal well-being and infant outcomes. We propose to assess 550 pregnant women: 350 pregnant women who report elevated levels of depressive symptoms and their infants (the depressed group), and 200 pregnant women who meet general inclusion criteria (regardless of depressive symptoms) and their infants (1100 total participants). Prior to the intervention, maternal measures will be collected. Then within the depressed group, half of the women will be randomized to receive approximately 8-weeks of brief interpersonal therapy (delivered during the first and second trimesters of pregnancy; approximately gestational weeks 12-20) after an initial engagement session. Maintenance sessions may be delivered as well, as agreed upon between the participant and the IPT therapist. The other half will receive enhanced usual care. After completion of the intervention, maternal measures will be collected longitudinally through 18 months postpartum for both the depressed and comparison participants. Maternal prenatal assessments will be conducted in person either at the University of Denver or University of Colorado Hospital at approximately 10, 24, and 34 weeks gestation, and by phone or by online survey at approximately 16 and 20 weeks gestation. In-person assessments may be offered to be completed by phone or online if the participant prefers to reduce participant burden. During prenatal visits, saliva, blood, and hair samples will be collected and assayed for stress markers including placental CRH, maternal plasma cortisol, and maternal hair cortisol. For participants recruited from the University of Colorado Hospital and Denver Health, the blood draw and saliva collection may occur at their regularly scheduled OB visit. Duration of time to delivery will be determined. For women who change their care provider location, or self-refer, options for home biomarker collection will be provided. Stool samples kits will be provided to understand gut microbiome of mother and infant, stool sample kits will be provided (for at-home collection) twice prenatally (approximately in the first and last trimester), and twice postnatally (approximately 6 and 18 months). Both infant and mother stool sample kits will be provided at the postpartum visits. Additionally, infant physiological stress regulation (hair cortisol), biomarkers and neurobehavior will be assessed after birth. At 2 months postpartum, we will interview the mother by phone or by online survey. Infant brain structure and function will be assessed within the first 3 postnatal months (corrected for preterm birth) by MRI scan in the Center for Innovation and Creativity (CINC) at the University of Colorado, Boulder (CU Boulder) or at the University of Colorado, Anschutz Medical Campus (CU Anschutz). During the MRI scan, the mother will complete a set of questionnaires. The mother and infant will then attend three postnatal lab visits at 6 months (range 5-9), 7 months (range 6-12) and 18 months (range 16-22) in the Neurodevelopmental Research Program at the University of Denver (DU). Maternal and child behavior will be evaluated at the postnatal lab visits, using standardized assessments/questionnaires; and infant physiological stress regulation will be evaluated (cortisol). At 12 months postpartum, the mother will complete a phone interview including a questionnaire and update to the clinical diagnostic interview (SCID). The clinical diagnostic interview (SCID) can also be updated at a later point if necessary. Participants who attend the 6, 7 and 18 month postnatal lab visits at DU, CU Anschutz, and/or CU Boulder will sign a supplemental DU consent form to remind them of what their postnatal participation will include, and to review their rights as a research participant.

We will also invite the father (or partner/secondary caregiver) to participate in a portion of the study to understand how paternal wellbeing may affect maternal wellbeing and infant outcomes. From here on, the term

fathers refer to the biological father as well as a partner (i.e. the person who will be providing the most infant care aside from the biological mother). Paternal measures will be collected longitudinally during pregnancy and the first year postpartum. Surveys for fathers will be conducted by phone or online survey at one-time point during the prenatal period and two time point between 0-12 months postpartum. Exact time points will be determined by the father's availability. Paternal participation does not in any way affect the mother's ability to participate fully in this project.

Recruitment.

We are recruiting two groups of women to participate in this study: 1) women with elevated depressive symptoms (the depressed group), and 2) a group comprised of any women who meet general inclusion criteria for the study (the comparison group). At their initial prenatal visit with their OB/GYN or midwife, all pregnant women at the recruitment sites at Denver Health (DH) and University of Colorado Hospital (UCH) complete the Edinburgh Postnatal Depression Scale (EPDS) as part of their standard care. Women who report an EPDS score of 9 or greater or are distressed based on clinical judgment are then referred to psychological support services (e.g. Promise Clinic). This study will use a cutoff of 9 and higher on the EPDS or are distressed based on clinical judgment to invite pregnant women to participate as part of our depressed group (presented to the mothers as "The Care Project: Helping Pregnant Moms and Babies"), as women scoring 9 or above are shown to have elevated levels of depressive symptoms. Women who meet general inclusion criteria (regardless of depressive symptoms) may be invited to participate as part of the comparison group. English-speaking women who meet criteria for either group will be approached about participation in the present study by the OB research team or an on-site Research Assistant (RA) at DH or UCH and who is directly supervised by Camille Hoffman, MD. The RA will explain the present study to the women. Those women who are interested in participation will be verbally screened for the following inclusion criteria: (1) 25 gestational weeks or less, (2), age between 18 and 45 years of age, (3) English speaking, and (4) singleton pregnancy. Women will also be screened and excluded from the study if they meet any of the following criteria: (1) current use of illicit drugs, (2) current use of methadone, (3) any major health conditions involving invasive treatments (e.g. dialysis, blood transfusions, chemotherapy), (4) experience of past or current psychotic symptoms or mania, (5) current engagement in cognitive behavioral therapy (CBT) or interpersonal therapy (IPT), (6) planning on terminating this current pregnancy, (7) is currently a prisoner. Those women who meet inclusion/exclusion criteria will then be invited and consented into the full study protocol. If participants are consented immediately, then the OB research team can complete the baseline visit, biosample collection or may schedule the first visit with the psychology research team. If the patient wants to think about the study, a member of the OB research team may ask the patient to sign a HIPAA A form and will follow up with the patient via a phone call. If the patient agrees to participate, the psychology research team (under the supervision of Dr. Elysia Davis) will conduct the baseline visit. For women who are recruited over the phone, they will be consented in person at the first study visit. Women who are referred to the study by participants or providers may call or email the Care Project team directly. A member of the Care Project team would then conduct preliminary screening (e.g. administer the EPDS, evaluate inclusion and exclusion criteria) to determine whether they are eligible to continue on in the study.

Once the mother is determined to be eligible and consents to the study, we will then ask her if we can contact the father of the baby (or secondary caregiver) and we will then provide an email link and/or contact the father by phone to see if they would like to participate in the paternal study.

Depression Treatment.

Within approximately one week after recruitment, mothers in the depressed group will be randomly assigned to receive either brief interpersonal therapy (IPT) or enhanced usual care (the standard of care at Denver Health and University of Colorado Hospital).

<u>Brief interpersonal therapy (IPT):</u> The intervention, known as "MOMCare" is a culturally relevant and

efficacious, collaborative care intervention that provides brief IPT via approximately 8 sessions after an initial engagement session, with maintenance sessions as agreed upon between the participant and the IPT therapist. Prior to beginning IPT sessions, a manualized pre-therapy engagement session is implemented to help resolve barriers to care (e.g., logistical, emotional, cultural). This engagement session is provided in-person at University of Denver, DH or UCH by a trained MOMCare provider (a licensed clinical social worker or a clinical psychology post-doctoral fellow). Thereafter, the same MOMCare provider who implemented the engagement session will continue as the provider of approximately 8 sessions of manualized brief IPT. These providers will follow women every week, tailoring sessions to the woman's preferences, as determined in the engagement session. Following completion of the first session, remaining sessions will occur both over the phone and in-person based on participant preference. Brief IPT includes approximately eight 50-minute individual sessions, and maintenance sessions (as agreed upon between the participant and IPT therapist). It focuses on psychoeducation and interpersonal skill building to decrease interpersonal conflict, increase interpersonal support and competence. Women are educated about the link between feelings and interpersonal interactions and are taught communication and interpersonal strategies that can be applied to different people in their lives. Brief IPT sessions will be audiotaped or video-taped and reviewed to rate adherence to and competence of IPT.

Enhanced usual care: Women in the depressed, enhanced usual care group will receive maternity support services (*MSS*), the usual standard of care at DH and UCH for pregnant women. Women who screen for possible depression diagnosis, (EPDS score of 10 or greater), are referred to psychological support services (e.g. Promise Clinic). Additionally, if women are randomized to receive enhanced usual care, we will provide them with NIH resource documents on depression.

Data Collection.

An overview of the study measures, including the timing of administration is presented in Table 1. Women in all study groups will participate in the same procedures for the remainder of the study protocol. An overview of the study measures for fathers, including timing is presented in Table 2. All father surveys will be self-report questionnaires in redcap and may be administered online, by phone or in person. The participant will choose their preferred method to reduce participant burden. Consent for fathers will be a postcard redcap consent before the survey.

Mothers may complete questionnaires at home, before or following an in-person visit. This will be done in an effort to shorten visit length and reduce participant burden. In cases in which the mother is scheduled for their first study visit and consent has not already been obtained by the recruiter, consent forms will be e-mailed to her. A researcher responsible for obtaining written informed consent will then call the participant once the consent have been received to review the consent form and answer any questions that the participants may have. If the participant agrees to participate, she will be asked to sign and date the consent form. She will then be sent a link to the questionnaires by the research team. None of the participants' questionnaire data will be used until written informed consent is obtained. If written consent is not obtained, any completed samples and/or questionnaires may be retained by the participant or be destroyed by the participant or a member of the research staff.

Maternal Mood, Questionnaires, and Descriptive Data (see Interview for Appendix and Questionnaire for Appendix).

Maternal report of depressive symptoms: We will use the 10-item Edinburgh Postnatal Depression Scale (EPDS; Cox et al, 1987) as our repeated measures assessment of depressive symptom severity (total of 9 maternal depressive symptoms assessments). The EPDS asks women about specific depressive symptoms over the previous week, with scores ranging from 0 – 30. Higher scores indicate more severe depressive symptoms. Depressive symptoms will also be evaluated using the Hopkins Symptom Checklist Depression Scale (SCL-20) and the Hamilton Depression Rating Scale (HAM-D). The EPDS, SCL-20, and HAM-D will be administered

during prenatal assessments before the intervention at approximately 10 gestational weeks and after initiation of the intervention at approximately 16, 20, 24, and 34 gestational weeks (5 prenatal assessments). We will continue with postnatal maternal assessments of her depressive symptoms at approximately 2, 6, 7, 12 and 18 months (5 postnatal assessments). EPDS will additionally be asked at the MRI visit (0-3 months). Additionally, the Patient Health Questionnaire-9 (PHQ-9) will be administered by the IPT therapists to monitor treatment effectiveness.

Maternal and paternal report of psychosocial functioning and other symptoms: Participants will be given measures throughout the study to assess their psychological health (e.g. rumination, stress, symptoms of anxiety), social relationships (e.g. social support, parental bonding), environmental stressors (e.g. chronic stress, discrimination, food security), life events (e.g. adverse childhood experiences, benevolent child experiences, unpredictability of childhood), life outlook (e.g. life satisfaction, mindfulness, self-compassion), attitudes towards the infant and pregnancy (e.g. attachment), acculturation, personality, IQ, sleep, physical activity, diet, and drug and alcohol use. For a full list of study questionnaires and the timing of measure administration, see Table 1 for mothers and Table 2 for fathers.

Clinical interview: Women will undergo additional assessment with a *Structured Clinical Interview for DSM-5 [Diagnostic and Statistical Manual, Revised], SCID).* This means that research-based psychological disorders will be determined using DSM criteria. In addition, women will be interviewed with the psychosis module, which asks about psychotic symptoms and features, including delusions and hallucinations. This is standard of care and will result in referral for clinical psychiatric evaluation. The SCID will be administered at the first prenatal visit and will be completed at another point if needed and at approximately 12 months postpartum and can be completed at another point if needed,and can be administered over the phone if necessary.

Maternal CBT usage and other psychiatric services use: To obtain data on maternal cognitive behavioral therapy (CBT) usage, we will assess mothers' reports of mental health services via reliable, valid items used and developed in the National Comorbidity Survey. Attitudes towards intervention will also be assessed using the Attitude Toward Intervention scale (ATI). Barriers to Access Care measure will be assessed.

Patient Satisfaction: at two points prenatally, pregnant women will be given the patient satisfaction questionnaire to understand their experience of the services received.

Infant Behavior Questionnaire: At the 6, 7 and 18-month visits, a valid parental report of infant behavior will be provided using Infant Behavior Questionnaire (IBQ-R).

Infant Feeding Questionnaire: At the fMRI visit, 2, 6 and 18-month visit, a questionnaire asking about infant feeding will be provided using a measure of infant feeding that the PI has used in multiple studies.

Infant Toddler Social Emotional Assessment (ITSEA): At the 18-month visit, a valid parental report of social emotional behavior will be provided using the ITSEA.

Participant background: Women will be interviewed to obtain demographic information including SES, household size and composition, marital status, occupation and education. We will apply structured interviews used in several NIH funded studies in our laboratory. This interview will be performed once prenatally and updated at 6 and 18-month visits. Fathers will be asked demographic information including SES, marital status, relationship to the baby (i.e. biological father or other), occupation, education, household size and composition. This will all be executed in redcap and will be administered either in person, phone or online depending on convenience for participant.

Biomarkers (Hormones and gut microbiome).

Maternal biomarkers and physical measurements: Maternal blood samples will be obtained 3 times at 6-10 weeks intervals depending on when she is recruited. For example, samples will be collected at approximately 10 gestational weeks (before intervention), at approximately 24 and 34 gestational weeks (after intervention), and at 6 and 18 months postpartum for assessment of hormones (e.g. plasma cortisol and placental CRH), as well as immune markers (e.g. CRP and IL-6), and telomere length. Blood samples will be collected by qualified health care personnel and/or a licensed phlebotomist at UCH, Denver Health or DU. Maternal blood samples will be collected (10 ml/draw). Plasma and serum will be decanted into polypropylene tubes and stored at –70ºC. Samples will be labeled with subject ID and time of sample collection recorded. We will also assess maternal height and weight during all lab visits. We will also collect maternal saliva via passive droll at all lab visits.

Maternal hair cortisol: A hair sample will be collected by a trained researcher at 3 times during gestation approximately 10, 24, and 34 weeks; and also at the birth visit and also at approximately 6 and 18 postnatal months. Several thin strands of hair will be carefully cut as close as possible to the scalp at the back of the head. The pooled hair sample, approximately half as thick as a pencil, will be stored until samples are assayed for cortisol using an established protocol (D'Anna-Hernandez et al, 2011). Additionally, mothers will also complete a contact sheet and a hair questionnaire designed to gather important information about maternal hair care and treatment that might influence the measurement of cortisol in hair. If the mother biosample isn't collected at University of Denver, UCH or Denver Health, it may be collected at a home visit.

Maternal microbiome (stool): A stool sample kit will be provided to women twice in pregnancy and twice postnatally (approximately first, third trimester and 6, 18 months postpartum). This will examine the bacteria that naturally reside in mother's intestines (gut). Differences in these natural bacteria have been linked to a variety of conditions, such as obesity, mood, temperament. It is hoped that we will begin to understand how different types of bacteria may affect maternal and child health. Briefly, women are instructed to wash their hands, collect stool from toilet paper with a swab, put the swab in a collection tube and stir for 1 minute and then discard the swab and close the tube tightly and then wash their hands again. The tube will then be mailed to the lab in a pre-paid envelop identified only by a kit ID number.

Infant hair cortisol: A hair sample will be collected from the infant after birth and at 6 and 18 months postpartum from a trained researcher. Mothers will hold their infant upright against their bodies and a trained member of the research team will collect approximately 50-100 strands of hair by carefully cutting hair from the nape of the neck as close to the scalp as possible. When inadequate hair is available at the nape of the neck, hair will be collected just superior to the nape of the neck. Scissors will be used and the procedure will be stopped if the infant becomes distressed. Participants' hair samples will be taped to and wrapped in untreated foil and stored until samples are assayed for cortisol using an established protocol (D'Anna-Hernandez et al., 2011). If the mother biosample isn't collected at University of Denver, UCH or Denver Health, the baby's hair may be collected at a home visit.

Infant biomarkers: During the infant's hospital stay, a nurse will perform the standard "heel stick" blood draw procedure on the infant (this is a standard hospital procedure done for every newborn and is **NOT** part of this research study). A member of our research team will observe the infant for 1 hour before and 1 hour after this procedure to record behavior, position, etc. If available, (the infant's heel is still bleeding), blood spots (up to 5 drops of blood) will be collected after the clinical procedure is completed. **We will never administer a heel stick or blood draw to infants for research purposes.** We will only collect this sample if the infant's heel is bleeding after the clinical procedure is completed. Samples may be assayed for hormone level and for telomere length (end

cap of chromosomes linked to cellular development). We will also collect saliva from the infant at the 6, 7 and 18-month lab visits via sterile swab. Samples will be collected 3 to 5 times over the course of each visit.

Infant gut microbiome (stool): A stool sample kit will be provided to parent twice in the first year of their baby's life, (6 and 18 months old approximately). This will examine the bacteria that naturally reside the baby's intestines (gut). Differences in these natural bacteria have been linked to a variety of conditions, such as obesity, mood, and temperament. It is hoped that we will begin to understand how different types of bacteria may affect maternal and child health. Briefly, the parent is instructed to wash their hands, collect stool from diaper with a swab, put the swab in a collection tube and stir for 1 minute and then discard the swab and close the tube tightly and then wash their hands again. The tube will then be mailed to the lab in a pre-paid envelop identified only by a kit ID number.

Maternal and Infant Health: Maternal and infant health will be assessed using an established questionnaire of health. This will be given at the baseline visit, in the third trimester as well as at the 6 and 18-month postnatal visit.

Maternal-child behaviors.

Maternal-child behavior will be assessed using a standardized observational protocol at 6, 7 and 18 months postpartum. Mothers will be asked to play with their child as they do at home for 10 minutes. Mother-child pairs will be video recorded and aspects of maternal behavior including sensitivity and responsiveness to infant cues and stimulation of child learning will be observed. Child behavior and mood will additionally be recorded.

Infant behavior.

Laboratory Temperament Assessment Battery: Infant behavior will also be assessed at the 6, 7 and 12-month visits, via a standardized developmentally appropriate laboratory paradigm implemented in our lab (protocol #2005-4756) and used in numerous published studies (e.g., Buss et al., 2004; 2005). This 10 to 20-minute evaluation will take place in child assessment rooms equipped with remote controlled cameras. The mother will be present for all episodes and the procedures will be monitored by a developmental psychologist. In this protocol the child is presented with a variety of novel situations including a mechanical toy that moves and makes noise, toys with flashing lights, toys that make noise, and an unfamiliar adult who is a member of the research team. Infants will additionally be presented with toys and activities that tend to elicit pleasure such as puppets or bubbles. Although these episodes sometimes elicit mild fear or wariness, most children do not become extremely distressed. Each episode is carefully monitored through remote controlled cameras and will be terminated if the child becomes distressed (e.g., cries for 20 seconds) or by maternal request. Each of the above episodes will be coded from video recordings to assess infant behavioral and emotional reactions. We have administered these procedures to hundreds of infants.

Eye tracking.
At approximately 6 and 18 months, infant attentional processing will be assessed using eye-tracking technology. An eye-tracker, a device used for measuring eye positions and eye movement, will record the infants face while he/she views photos of faces showing a range of emotions (happy, angry, sad, and neutral). Infants will be able to passively view pairs of these faces on the screen for 5 seconds each. Time spent viewing each face will be recorded to explore how infants attend to emotion and how this might be influenced by maternal characteristics. In the second eye-tracking task, infant cognitive control and attention regulation will be assessed. The infant will be viewing a screen that shows targets (i.e. a purple x, a yellow ring, or a red square), to explore how infants orient to spatial location as the targets will appear in different areas of the screen. The infant will be sitting on the mother's or research assistant's lab, or in a high chair during these assessments (depending on the preference

of the parent and/or infant). These are standardized measures that are routinely administered to infants and have no known risks. Each task is carefully monitored by an experimenter, seated in the room and behind an occluder, and will be terminated if the child becomes distressed (e.g., cries for 20 seconds) or by maternal request.

Electroencephalography (EEG) recording.
At approximately 7 and 18 months, infant EEG will be recorded using a 64-channel EGI sensor net and NetStation software. The sensor net is carefully placed on the infant's head and held in place with an elasticated strap beneath the chin. Net application and EEG recording will be performed by trained experimenters. Once proper electrode placement and data transmission has been confirmed, the infant will be able to passively view photographs of faces showing a range of emotions (e.g. happiness, sadness, anger, fear, and neutral) or shapes presented on a computer screen. Next, the infant will passively view videos or distracting stimuli (e.g. watching a research assistant blow bubbles). EEG data collected during these tasks will be used to examine group-level differences in the development of attention, memory, and emotion reactivity. For example, amplitudes of ERP components related to attentional orienting (e.g., Nc) will be averaged across infants of mothers in the treatment group and compared to those averaged across infants of mothers in the enhanced usual care group to examine potential group differences in the development of attentional control (see Parker & Nelson, 2005 for a similar approach to the use of EEG data). An individual's data will not be compared to group norms nor provided as feedback to participants. The infant will be sitting on the mother's lap or securely in a highchair during these assessments. These are safe, standardized paradigms that are routinely administered to infants and have minimal risks. Risks include possible, minor discoloration on the infant's skin after the sensor net is removed; this will typically go away within 20 minutes of net removal. Each task is carefully monitored by an experimenter, seated in the room and behind an occluder, and will be terminated if the child becomes distressed. Additionally, the mother may choose to stop the assessment at any time. No medical diagnosis will be made based on the infant EEG recordings.

Medical chart review.
Maternal pregnancy and birth outcome medical records: Maternal prenatal history (e.g., number of prenatal visits, pregnancy complications, gravidity, parity, etc.), and birth outcome and the neonatal course (gestational age at birth, route of delivery, birthweight, and Apgar scores at 1- and 5-minutes) will be obtained through review of the participant's (mother and infant) medical records (see appendix for type of information that will be abstracted from medical records).

Neuroimaging.
Eligible mothers and infants will visit the Center for Innovation and Creativity (CINC) at the University of Colorado, Boulder, or the University of Colorado, Anschutz Medical Campus when their baby is less than three months old. Both neuroimaging centers are equipped with a 3 Tesla MRI scanner, which will be used in this study, as well as multiple behavioral testing rooms, and a private waiting room furnished ideally for families with infants (a changing table, a crib, a rocking chair, and small toys). The visits will typically occur around a baby's regular afternoon nap time or the infant's regular evening sleep time during weekdays or weekends.

Infants will be screened for any metal (e.g., coins, jewelry) on their body. If mothers opt to stay in the MRI room, mothers will also be screened for any metal on their body and be provided with a pregnancy test (mothers will be informed of this during scheduling so that they may plan for an additional care provider to be present). If the mother is ineligible (e.g. currently pregnant) or if she chooses to not be in the scanning room, a father or another family member is encouraged to accompany the mother and infant and opt to stay in the MRI room. In all cases a trained member of the research staff will be present in the scan room.

The scanner room will be equipped with a chair for mothers to sit in to help the infant to fall asleep. If mother permits, a trained researcher will also offer to help. Once the infant falls asleep, the researcher and a MRI technician will safely place the infant on memory foam (MRI-safe) on the scanner bed. Ear plugs and pediatric

headphones will be placed to cover the infant's ears to reduce noise levels. To further reduce the noise level and prevent infants from waking up due to the noise during the scan, a removable sound-insulating foam insert may be placed on the scanner.

The scanner is operated by a professional MRI technician at all times. A trained researcher will remain inside the scanning room at all times in case the infant wakes up during the scan. Mothers or an alternative care provider are also invited to remain in the imaging suite during the scan (if they pass MRI safety screening). The researcher will explain to the mother that she can stop the scan at any time if there are any questions or concerns regarding instructions and procedures, or if they are in any way feeling uncomfortable. If the infant wakes up during the scan, scanning will be immediately stopped and the infant will be brought out of the MRI scanner. Mothers will be allowed to comfort the infant and try to have the infant fall back asleep. If the mothers think the infant would not return to sleep, they will be encouraged to schedule another MRI session. The total duration of the MRI session is expected to be about 90 minutes (60 min. for MRI scanning and 45 min. for falling asleep, setting up and wrapping up. The total duration of the MRI visit will be approximately two hours. Families will also receive images of the infants' brain. Mothers will be told that that the brain images they receive are for personal use only and cannot be used for clinical evaluations. Providing the family with images of the infant's brain taken during the MRI lab visit is standard procedure for MRI research and increases the family's sense of contributing to science.

If a potential brain irregularity is observed during the MRI, the scan will be assessed by a professionally licensed neurologist or radiologist. In the event that the professional finds the observation to be potentially medically notable we will report the information to the child's parents and the child's primary care provider. The neurologist has extensive experience talking with parents about abnormal findings, and does so in a way that is non-alarmist and reassuring. If referral to a neurologist is deemed necessary, we will assist the parent of that child with that referral

<u>Neuroimaging measures:</u> A structural scan (approx. 9 minutes) will be done to collect structural images of the brain and map the functional brain activity on the structural images. A diffusion tensor imaging (DTI) scan will assess infant white matter tracts in the brain (approx. 15 minutes). A resting scan (approx. 10 minutes) will assess brain functional connectivity while resting in the scanner (this is performed twice).

Data Analysis.

We will test our main aims and hypotheses regarding reduction in maternal depressive symptoms and its effect on infant mechanisms via two complementary approaches. (1) <u>Between groups</u> (IPT vs. MSS enhanced usual care): Based on prior work and our meta-analysis we expect that the IPT group will show a significant reduction in depressive symptoms compared to MSS. Further, between group analyses will test our hypotheses that offspring of women in the IPT group will show reduced potential threat/"anxiety" and greater cognitive (effortful) control compared to infants of women in MSS group. (2) <u>Within subject</u> analyses will characterize individual differences in mothers and fathers in the change in depressive symptoms over time and strength of association with infant outcomes. Specifically, the infants of mothers whose prenatal depressive symptoms decrease to a greater extent will show larger reductions in potential threat/"anxiety" and greater cognitive (effortful) control. (3) We will conduct limited, focused exploratory analyses to look at moderators and mediators of the association between maternal depressive symptoms and infant outcomes. (4) Women who receive CBT or IPT (outside of randomization), or psychotropic medication use will be excluded from all analyses. (5) Additionally, we will assess how participants in the IPT group compare to the comparison group following completion of treatment. Analyses will be performed with consultant Stern, who has expertise with complex longitudinal data.

<u>Preliminary analyses:</u> Prior to initiation of primary analyses we will determine whether groups differ in potential

covariates (e.g., sociodemographic, birth outcome (including GAB), obstetric factors) or whether there is differential attrition between the IPT and MSS groups. Propensity score matching would be used to adjust if there are group differences or differential attrition. Other psychiatric service usage, besides CBT or psychotropic medication which are exclusions, will be examined as covariates in analysis.

1. Between groups. We will use a mixed effects model (MEM) framework, where we summarize change over time for (a) maternal depressive symptoms (EPDS) and (b) infant outcomes of risk mechanisms, respectively, per intervention arm. Contrasts will be examined through the use of response features to address any potential complex patterns of change (e.g., nonlinear). Within the MEM framework, we will contrast the two prevention conditions (IPT vs. MSS). The MEM framework accommodates the clustering of subjects within intervention groups and will be used to examine both sets of dependent measures: (a) maternal depressive symptoms (EPDS) and (b) infant outcomes of risk mechanisms at birth, 6 and 12 months.

2. Within subject analyses. To more directly test our model that greater decreases in maternal symptoms over time are the effect of IPT, and these symptom reductions, in turn, result in improved infant outcomes, we will use a mixed effect structure via a 2-level multilevel model (HLM) to examine change in maternal symptom trajectories (Level 1) as a function of intervention condition (Level 2). These analyses will test the hypothesis that greater reductions in depressive symptoms trajectories over time will relate to improved infant outcomes, after taking into account the between groups effect of intervention. Next, we will implement a causal mediation approach, to account for the change in the respective symptom decreases leading to subsequent change in infant risk outcomes. Causal mediation models adjust for potential unmeasured confounding variables in this sequential lagged process (prior maternal symptom change leading to subsequent infant outcome change).

3. Exploratory Analyses. We will consider: a) plausible mechanisms, b) the independent and joint role of prenatal and postnatal influence, and c) potential moderators.

3a. Potential mechanisms by which reducing maternal depressive symptoms reduces infant risk mechanisms: Using the 2-level multilevel model described above (A.2), we will test the exploratory hypothesis that reducing maternal depressive symptoms will alter maternal cortisol and placental CRH trajectories, as part of a partial mediation (or indirect path) model, which in turn will effect change in infant risk mechanisms.

3b. Prenatal vs. postnatal influences: To determine whether beneficial effects of IPT on infant pathophysiology work through prenatal mechanisms and/or via joint effect with postnatal processes (e.g., parenting, postnatal depressive symptoms), we will employ two strategies. First, infants will be evaluated 24-hours after birth (heel stick; NNNS). Any intervention effects on these neonatal functioning measures are not likely due to postnatal experiences. Second, maternal behavior and postnatal depressive symptoms will be entered into predictive models (1) to identify the benefit of IPT after considering postnatal influences and (2) to determine if there are additive and/or synergistic influences of prenatal and postnatal experiences.

3c. Examination of potential moderators: All of the analytic procedures described above enable inclusion of predictors, which can be modeled as main effects or interactive terms to assess the impact of moderators (e.g., psychosocial measures such as anxiety, stress, and social support; demographic measures such as maternal age, SES, race/ethnicity; infant measures such as infant sex, GAB, birth weight) as predictors of outcome (i.e., main effects) or differential predictors of outcomes (i.e., interaction effects). For moderated mediation analyses, these moderator variables will be assessed as potential interacting factors of each leg of the pathway model.

4. <u>*Father analysis*</u>: To determine the trajectory of paternal depression from pregnancy through twelve months postpartum, a repeated measures ANOVA model will be used. A HLM model will be used to determine predictive variables contributing to father's depression and what risk and resiliency factors may affect depressive symptoms. Finally, a dyadic partner effects model will be used with maternal depressive symptoms to predict infant pathophysiological outcomes.

5. *Microbiome analysis:* Microbiome data will be used to determine: 1) associations between maternal mood, infant temperament, and gut microbiome over time; 2) Effects of infant exposures (e.g., C-section, breastfeeding, antibiotics) on infant gut microbiome.

Table 1. *Overview of maternal and infant study measures.*

See attachment –Care Project Measures Checklist

Notes: Dots represent optimal timing of administration. Measures may be shifted to a different visit based on visit timing and participant availability. Additionally, the Patient Health Questionnaire-9 (PHQ-9) will be administered by the IPT therapists at each MOMCare session to monitor treatment effectiveness.
* Relationship Question (RQ) may also be administered by IPT therapists.
** corrected for preterm birth

Table 2: *Overview of paternal study measures*

**Measure**	**Prenatal**	**Postnatal**	
	**T1 (0 – 20wks)**	**0-6 months**	**6-12 months**
Demographics	•		
What is the experience of the birth like (qualitative)		•	
Role of the Father Questionnaire	•		•
EPDS	•	•	•
EPDS about mom	•	•	•
LEC	•		
ACE (with age breakdowns)	•		
PCL-5	•	•	
BCEs (with age breakdowns)	•		
FFMQ-SF (Mindfulness)	•		
Mindful Parenting		•	•
Self-Compassion	•	•	•

TESTING PROJECT HYPOTHESES

Data Analysis

We will test our main aims and hypotheses regarding reduction in maternal depressive symptoms and its effect on infant mechanisms via two complementary approaches. (1) Between groups (IPT vs. MSS enhanced usual care): Based on prior work and our meta-analysis we expect that the IPT group will show a significant reduction in depressive symptoms compared to MSS. (2) Within subject analyses will characterize individual differences in the change in depressive symptoms over time. (3) Women who receive CBT or IPT (outside of randomization), or psychotropic medication use will be excluded from all analyses. Analyses will be performed with consultant who has expertise with complex longitudinal data.

Preliminary analyses:

Prior to initiation of primary analyses we will determine whether groups differ in potential covariates (e.g., sociodemographic, birth outcome (including GAB) obstetric factors) or whether there is differential attrition between the IPT and MSS groups. Propensity score matching would be used to adjust if there are group differences or differential attrition.[189, 190] Other psychiatric service usage, besides CBT or psychotropic medication which are exclusions, will be examined as covariates in analysis.

1. Between groups: We will use a mixed effects model (MEM) framework, where we summarize change over time for maternal depressive symptoms per intervention arm. Contrasts will be examined through the use of response features to address any potential complex patterns of change (e.g., nonlinear). Within the MEM framework, we will contrast the two prevention conditions (IPT vs. MSS). The MEM framework accommodates the clustering of subjects within intervention groups.

2. Within subject analyses: To more directly test our model that greater decreases in maternal symptoms over time are the effect of IPT, we will use a mixed effect structure via a 2 level multilevel model (HLM) to examine change in maternal symptom trajectories (Level 1) as a function of intervention condition (Level 2). These analyses will test the hypothesis that greater reductions in depressive symptoms trajectories over time.

Statistical Power:

Power analysis for intervention. This study uses brief IPT, a well-established, efficacious treatment for prenatal depression. We conducted power analyses to ensure that we had a sufficiently powered sample size to detect differences in maternal depressive symptoms between groups (Brief IPT and MSS as control). Using d = .55 as our effect size estimate (per our meta-analysis on Brief IPT trials with antenatal depression) with a sample size of 128 women per group, we estimated power to be 99% to detect a medium effect size of .50.

Figure 6

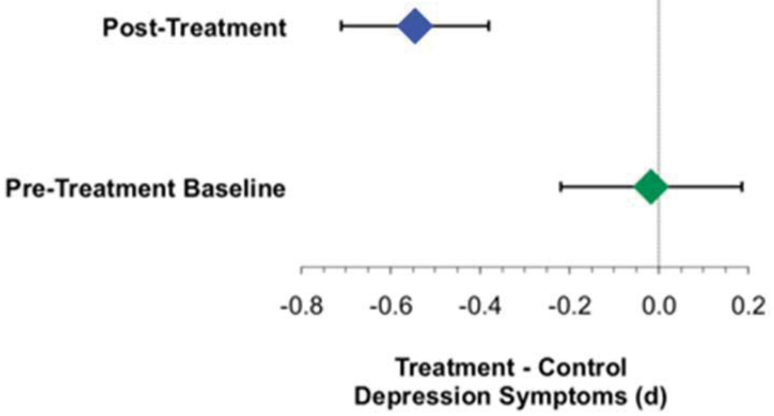

For the within-subject portion of our data analyses using multilevel modeling (HLM), statistical power is based on the methods described by Raudenbush et al[192-194] and implemented with the Optimal Design HLM Software package. Power calculations were estimated for a two-level clustered design, which addresses the repeated measures per subject and with subjects nested within intervention arm. We set parameter values as follows: a) within subject correlation of 0.35 for the repeated measures, and b) an effect size (d) as indicated above based on prior research. With our lowest anticipated number of subjects included in the analysis (N=219), we estimated power of 96.1% to detect a medium effect size (d=0.5).
