# Supplementary material for: Effect of Brief Interpersonal Therapy on Depression During Pregnancy: A Randomized Clinical Trial
Source: JAMA Psychiatry. 2023 Apr 19;80(6):539–47. doi: 10.1001/jamapsychiatry.2023.0702 (PMC10116385; doi:10.1001/jamapsychiatry.2023.0702)
Supplement: Supplement 2. — eMethods 1. Additional details on CONSORT diagram for exclusions eMethods 2. Adherence and fidelity ratings eFigure. Depiction of general Care Project RCT timeline, with the time points and study elements occurring when they tended to happen on average for modal participants eAppendix. MOMCare Vignette eResults 1. Change over time details eResults 2. Moderation eResults 3. Therapist or site effects eResults 4. COVID differences eResults 5. Sensitivity analyses for psychiatric medication use [file jamapsychiatry-e230702-s002.pdf]

Supplemental Online Content

Hankin BL, Demers CH, Hennessey E-MP, et al. Effect of brief interpersonal therapy on depression during pregnancy: a randomized clinical trial. *JAMA Psychiatry*. Published online April 19, 2023. doi:10.1001/jamapsychiatry.2023.0702

eMethods 1. Additional details on CONSORT diagram for exclusions
eMethods 2. Adherence and fidelity ratings
eFigure. Depiction of general Care Project RCT timeline, with the time points and study elements occurring when they tended to happen on average for modal participants
eAppendix. MOMCare Vignette
eResults 1. Change over time details
eResults 2. Moderation
eResults 3. Therapist or site effects
eResults 4. COVID differences
eResults 5. Sensitivity analyses for psychiatric medication use

This supplemental material has been provided by the authors to give readers additional information about their work.

© 2023 Hankin BL et al. *JAMA Psychiatry.*

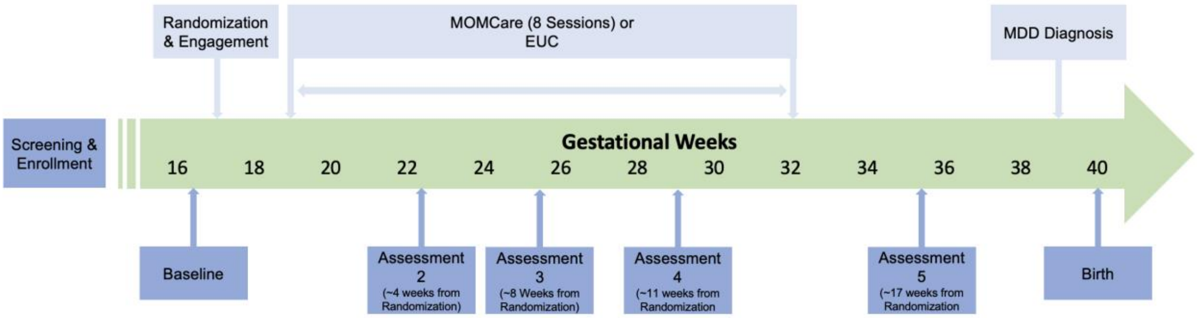

eFigure. Depiction of general Care Project RCT timeline, with the time points and study elements occurring when they tended to happen on average for modal participants. Time points of assessment were not fixed to explicitly specific times (weeks GA or since randomization); rather this figure presents a prototypically accurate depiction of study flow with flexibility and variability around modal times.

eAppendix. MOMCare Vignette

A key element of IPT is the identification of a present and specific problem area; the four traditional IPT problem areas include role transitions, role disputes, interpersonal deficits, and grief (Weissman, Markowitz, & Klerman, 2018). Spinelli & Endicott (2003) established “complicated pregnancy” as a fifth problem area, considered to be a subtype of role transition, specific to some pregnant individuals because many of the unique issues associated with pregnancy that can contribute to mental health difficulties including depression and anxiety are not adequately covered by the original problem areas. Patients whose focal concerns relate to the problem area of complicated pregnancy present with problems specific to pregnancy such as unplanned or unwanted pregnancy, medical concerns associated with pregnancy, obstetric complications, and congenital or other developmental concerns.

Most participants in the Care Project who were randomized to receive IPT were conceptualized to have complicated pregnancy as their primary problem area. The second-most common problem area in the present sample was role transition, which was commonly characterized by the transition to first-time parenthood. To exemplify typical IPT treatment as delivered through the Care Project we present the following case Vignette.

Case Vignette – Ana *(name and identifying features changed to protect patient privacy)*

Ana is a 22-year-old cisgender woman (she/her), first-time parent and 16 weeks pregnant, who identified as Hispanic. Ana worked full-time in retail and lived with her biological parents and two sisters. She was recruited to participate in the Care Project after she reported elevated symptoms of depression at an obstetrics appointment suggesting moderately severe depression. During her engagement session, Ana reported feeling overwhelmed and scared about her pregnancy with low mood, poor sleep, and worries about how the stress she was experiencing might impact her baby’s development. Stressors Ana endorsed included an

© 2023 Hankin BL et al. *JAMA Psychiatry.*

unplanned pregnancy, fears related to pregnancy and childbirth, conflicts with family members and a past romantic partner, low social support, a family history of substance abuse, and concerns about father of the baby's (FOB)'s ability to support her due to his ongoing recovery from substance abuse. Ana described several traumatic experiences in her past as well as a history of depression and anxiety. She endorsed barriers to treatment, including unreliable transportation, an unpredictable work schedule, and family denial of her trauma history. She reported a negative past therapy experience in which she sought treatment for depression and her provider was "dismissive", didn't listen to her, was very "doctor-like", and "just wanted to medicate [her] to fix her problems." Ana and the therapist collaboratively addressed barriers to care, with attention to Ana's need for scheduling flexibility and the option for telehealth sessions due to transportation and scheduling challenges. The therapist affirmed Ana's dedication to becoming a healthy, loving, and supportive parent, and honored her self-efficacy in deciding whether she wanted to participate in treatment.

Initial IPT sessions focused on assessment of Ana's biopsychosocial and cultural history, creating a timeline of Ana's experiences with depression and anxiety, completing the interpersonal inventory, and identifying an IPT problem area. Ana's mood worsened and anxiety increased after she learned she was pregnant, and these symptoms were amplified by financial stress, Ana's existing caregiving responsibilities for FOB and other family members, and ongoing interpersonal conflicts. The therapist and Ana explored both stressful and supportive relationships in Ana's life and determined that "complicated pregnancy" best fit Ana's presenting concerns due to her pregnancy being unplanned, limited social and financial resources, ongoing interpersonal stress associated with substance abuse by many important people in Ana's life, and her fears and worries about pregnancy and childbirth.

Treatment focused on encouraging Ana to explore and express her feelings around the transition to becoming a parent, acknowledging what was being lost from her life before parenthood, building social supports, connecting with her baby, and learning ways to ask for what she wanted and needed. Ana's homework often involved taking time to care for herself, asking FOB to support her in preparing for baby's arrival, and setting boundaries with stressful family members. As treatment progressed Ana's goals for treatment emphasized feeling better, reducing stress in her life, strengthening supportive relationships, and connecting with FOB to prepare for the birth of their baby. To maintain treatment engagement and participation the therapist regularly communicated with Ana by text and offered flexible scheduling with the option for telehealth sessions.

Ana completed a full course of IPT (8 sessions) and her depression score dropped to a level suggesting mild depression (maintained primarily by symptoms also associated with the third trimester of pregnancy including fatigue and poor sleep). Ana gave birth to a healthy full-term baby girl, immediately bonded with her baby, and continued bi-weekly and then monthly maintenance IPT sessions for the first year postpartum. She reported that IPT was very helpful to her, noting that she learned to recognize and communicate how she was feeling, gained comfort receiving care and asking for help, and learned how to maintain relationships with stressful family members while protecting her own and her child's wellbeing. She navigated numerous stressors in her first year postpartum without recurrence of depression, including two moves, a job transition, and a family member's substance abuse relapse. Despite these challenges, Ana maintained steady employment and secure housing, strengthened her relationship with FOB and several supportive friends, managed relationships with family members, and experienced parenting joy and a close connection with her daughter.

Weissman, M.M., Markowitz, J.C., & Klerman, G.L. (2018). *The Guide to interpersonal psychotherapy: Updated and expanded edition.* Oxford University Press.

© 2023 Hankin BL et al. *JAMA Psychiatry.*

Spinelli, M.G., & Endicott, J. (2003). Controlled clinical trial of interpersonal psychotherapy versus parenting education program for depressed women. *American Journal of Psychiatry, 160*(3), 555-562.

eMethods 1. Additional details on CONSORT diagram for exclusions.

Of the 657 excluded, 181 declined, 374 could not be contacted successfully, 100 did not show for eligibility assessment despite multiple recontacts, and 2 had spontaneous abortion (SAB) prior to eligibility assessment.

Of the 47 excluded, 28 did not meet eligibility criteria: 1 used illicit substances, 10 reported symptoms of psychosis or mania, 6 received psychiatric diagnoses that required additional care (eating disorder and dissociative identity disorder) or were deemed high risk level for study during pregnancy (major health conditions requiring invasive medical treatments), 6 were unable to use virtual communication for the study during COVID, 2 were having twins, 1 was receiving CBT, and 2 did not meet depression criteria.
For the 19 with other reasons for exclusion, 7 did not show up to a baseline assessment and 12 did not show up for initial visit for randomization.

eMethods 2. Adherence and fidelity ratings.

IPT sessions were audio recorded for supervision and for later adherence and fidelity ratings.

Fidelity ratings were provided by Nancy Grote and Mary Curran, who did the MOMCare trainings and supervision (NG developed MOMCare, and MC has been co-author on many Brief IPT papers and projects). They listened to recorded sessions and provided the fidelity ratings using IPT adherence checklists developed by NG for MOMCare trials. The IPT adherence checklist is a 27 item Therapy Rating Scale modeled on the scale developed by De Rubeis et al. for use in the Treatment of Depression Collaborative Research Program.

The IPT adherence checklists were rated by these two IPT experts to ensure that the IPT model was being followed with fidelity. Questions on the fidelity check list ask how well the therapist did the following:
Assessment and mood rating, check on homework, complete interpersonal inventory, case formulation, contract, use IPT specific strategies (including encourage release of affect, conduct communication analysis, problem solve), collaboratively assign homework, summarize session, elicit feedback, connect current session to prior sessions, affirm strengths, use summaries, collaborate with patient, pay attention to affect, and maintain positive therapeutic alliance.

All participants receiving IPT had at least one session reviewed for fidelity. Different proportions of sessions were reviewed across the course of IPT: 25% of engagements sessions, 25% of IPT 1st session, 31% of IPT 2nd session, and 41% of remaining active sessions.

© 2023 Hankin BL et al. *JAMA Psychiatry.*

RESULTS and DATA ANALYSIS DETAILS

eResults 1. Change over time details.

Our mathematical assessment showed change over time was best described by a linear trajectory. We fit HLM models with random intercept and random slope term with an unstructured covariance structure which allows the subject-specific intercept to be correlated with the subject-specific slope. Additionally, similar to the classic Gibbons et al. (1993) implementation of HLM to psychiatric data, on top of the specified random effects, we fit an autoregressive error structure for the residuals.

eResults 2. Moderation.

With the HLM models our primary effect of interest is the intervention interaction with time; therefore, indicating “differential rate of change over time”. For the moderation models, we augmented our original models with the primary focus on the three-way interaction of the proposed moderator by intervention by time effect. Potentially statistically significant interactions were interpreted by derived Slope estimates per intervention at each level of categorical moderators (i.e. MDD) or for high and low values of continuous moderators (i.e. gestational age) defined as ±1 standard deviation above and below the mean (Holmbeck, 1997; Aiken & West, 1991).

Baron, R. M. and Kenny, D.A. (1986). The moderator-mediator variable distinction in social psychological research: Conceptual, strategic, and statistical considerations, *Journal of Personality and Social Psychology,* 51, 1173-1182.

Holmbeck GN (1997). Towards terminological, conceptual, and statistical clarity in the study of mediators and moderators: examples from child clinical and pediatric psychology literature, *Journal of Consulting and Clinical Psychology*, 65, 599-610.

Aiken, L.S. & West, S.G. (1991). *Multiple regression: Testing and interpreting interactions.* Newbury Park, CA: Sage.

eResults 3. Therapist or site effects.

Four therapists provided intervention in MOMCare, and there were three main providing clinical sites (2 medical centers and third as other smaller locations from which participants enrolled).

We conducted additional analyses to nest participants within therapists and then within clinics.

We augmented our original 2-level model to a 3-level model. To test significance of therapist effects, we follow the procedures described by Verbeke and Molenbergh (2000), who provide an outline we parallel for testing the need for therapist and clinic random effects. Two issues we must consider are (i) the respective estimated variance components to go to 0 and (ii) significance testing is assessed by a mixture of a chi-square distribution. If classical null distribution would be used, all p-values would be overestimated; therefore, the null hypothesis would be accepted too often and a simplified covariance structure would be modeled, which may impair inferences (Altham, 1984). The 3-level model, revealed no significant therapist effects for EPDS ($\chi^2_{0:1} = 0.33$, P=0.64) and no significant therapist effect for SCL-20 ($\chi^2_{0:1} = 0.02$, P=0.90). Similarly, the augmented 4-level model revealed no significant clinic effects for EPDS ($\chi^2_{0:1}$ =0.72 , P=0.60) and no significant clinic effects for SCL-20 ($\chi^2_{0:1}$ =0.71 , P=0.60).

© 2023 Hankin BL et al. *JAMA Psychiatry.*

Due to the non-significance of either therapist or clinic effect for both EPDS and SCL-20, these therapist and clinic factors were dropped from further analyses as reported in primary text.

Altham, P.M.E. (1984). Improving the precision of estimation by fitting a mode. Journal of the Royal Statistical Society, Series B, 46, 118-119.

Verbeke, G., & Molenberghs, G. (2000). Linear Mixed models for Longitudinal Data New York: Spinger-Verlag.

eResults 4. COVID differences.

For the **SCL-20**, 42.3% of the assessments were on or after 3/10/2020, corresponding to the COVID shutdown. Whether the overall effect was moderated by the onset of COVID yielded a non-significant effect t=-0.77, *P*=0.41.

Overall, we reported an on-average difference of 0.402 (se=0.093) t = 4.31, *P*<0.0001 in the rate of change per week (*d*=0.57, 95%CI 0.22-0.91). Prior to onset of COVID, we see an on-average difference of 0.466 (se=0.143) t=3.26, *P*=0.001 in the rate of change per week (*d*=0.52, 95%CI 0.20-0.83). Post the onset of COVID, the on-average difference in the rate of change reduces to 0.294 (se=0.110) t=2.67, *P*=0.008 (*d*=0.50, 95%CI 0.13-0.86).

For the **EPDS**, 42.1% of the assessments were on or after 3/10/2020, corresponding to the COVID shutdown. Whether the overall effect for EPDS was moderated by the onset of COVID yielded a non-significant effect t=-0.31, *P*=0.75.

Overall, we reported an on-average difference of 0.095 (se=0.035) t = 2.73, *P*=.007 in the rate of change per week (*d*=0.40, 95% CI 0.06-0.74). Prior to onset of COVID, we see an on-average difference of 0.087 (se=0.043) t=2.02, *P*=0.045 in the rate of change per week (*d*=0.32, 95%CI 0.01-0.63). Post the onset of COVID, the on-average difference in the rate of change reduces to 0.069 (se=0.046) t=1.51, *P*=0.13 (*d*=0.28, 95%CI -0.08-0.64)

eResults 5. Sensitivity analyses for psychiatric medication use

Whether the overall effect of decreased **SCL-20** within IPT group was moderated by medication use yielded a non-significant effect t=0.21, *P*=0.76. Overall, we reported an on-average difference of 0.402 (se=0.093) t = 4.31, *P*<0.0001 in the rate of change per week (*d*=0.57, 95%CI 0.22-0.91). In patients not using medication, we see an on-average difference of 0.386 (se=0.104) t=3.72, *P*=0.0003 in the rate of change per week (*d*=0.55, 95%CI 0.20-0.90). In patients using medication, the on-average difference in the rate of change reduces to 0.262 (se=0.198) t=1.34, *P*=0.18 (*d*=0.38, 95%CI -0.19-0.93).

Whether the overall effect of decreased **EPDS** within IPT group was moderated by medication use yielded a non-significant effect t=-0.60, *P*=0.55. For the EPDS we reported an on-average difference of 0.095 (se=0.035) t = 2.73, *P*=.007 in the rate of change per week (*d*=0.40, 95% CI 0.06-0.74). In patients not using medication, we see an on-average difference of 0.144 (se=0.025) t=2.83, *P*=0.005 in the rate of change per week (*d*=0.42, 95%CI 0.12-0.71). In patients using medication, the on-average difference in the rate of change reduces to 0.042 (se=0.066) t=0.63, *P*=0.53 (*d*=0.18, 95%CI -0.38-0.73).

© 2023 Hankin BL et al. *JAMA Psychiatry.*
