# Supplementary material for: Effect of Brief Interpersonal Therapy on Depression During Pregnancy: A Randomized Clinical Trial
Source: JAMA Psychiatry. 2023 Apr 19;80(6):539–47. doi: 10.1001/jamapsychiatry.2023.0702 (PMC10116385; doi:10.1001/jamapsychiatry.2023.0702)
Supplement: Supplement 3. — Data sharing statement [file jamapsychiatry-e230702-s003.pdf]

Data Sharing Statement

Hankin. Effect of Brief Interpersonal Therapy on Depression During Pregnancy. *JAMA Psychiatry*. Published April 19, 2023. doi:10.1001/jamapsychiatry.2023.0702

Data
Data available: No

Additional Information
Explanation for why data not available: There still exists confidential and identifiable patient information at the individual patient data level. We can share data per NIMH Data Sharing Rules for the funding R01 with reasonable requests.
